# Comparison of tissue pressure and ablation time between the LeVeen and cool-tip needle methods

**DOI:** 10.1186/1476-5926-5-10

**Published:** 2006-12-21

**Authors:** Makoto Nakamuta, Motoyuki Kohjima, Shusuke Morizono, Tsuyoshi Yoshimoto, Yuzuru Miyagi, Hironori Sakai, Munechika Enjoji, Kazuhiro Kotoh

**Affiliations:** 1 Department of Medicine and Bioregulatory Science, Graduate School of Medical Sciences, Kyushu University, Japan; 2 Department of Gastroenterology, National Hospital Organization Kyushu Medical Center, Fukuoka, Japan

## Abstract

**Background:**

Radio frequency ablation (RFA) has been accepted clinically as a useful local treatment for hepatocellular carcinoma (HCC). However, intrahepatic recurrence after RFA has been reported which might be attributable to increase in intra-tumor pressure during RFA. To reduce the pressure and ablation time, we developed a novel method of RFA, a multi-step method in which a LeVeen needle, an expansion-type electrode, is incrementally and stepwise expanded. We compared the maximal pressure during ablation and the total ablation time among the multi-step method, single-step method (a standard single-step full expansion with a LeVeen needle), and the method with a cool-tip electrode. Finally, we performed a preliminary comparison of the ablation times for these methods in HCC cases.

**Results:**

A block of pig liver sealed in a rigid plastic case was used as a model of an HCC tumor with a capsule. The multi-step method with the LeVeen electrode resulted in the lowest pressure as compared with the single-step or cool-tip methods. There was no significant difference in the ablation time between the multi-step and cool-tip ablation methods, although the single-step methods had longer ablation times than the other ablation procedures. In HCC cases, the multi-step method had a significantly shorter ablation time than the single-step or cool-tip methods.

**Conclusion:**

We demonstrated that the multi-step method was useful to reduce the ablation time and to suppress the increase in pressure. The multi-step method using a LeVeen needle may be a clinically applicable procedure for RFA.

## Background

Hepatocellular carcinoma (HCC) is one of the most common cancers worldwide. Most of HCC patients suffer from virus-induced liver injury and most have underlying liver cirrhosis [1]. Percutaneous ethanol injection therapy (PEIT) has been used widely for the treatment of unresectable HCC [2]. Many reports showed that the efficacy of PEIT for small HCC tumors was comparable to that of hepatic resection; however, PEIT demands multiple sessions to achieve complete necrosis, resulting in protracted hospitalization [3]. Furthermore, many patients suffer from local recurrence after PEIT, which is attributable to intra-tumor septa that prevent the injected ethanol from infiltrating the entire tumor [4,5]. We reported that local recurrence after PEIT should be prevented as much as possible because it is one of the most important negative prognostic factors for HCC patients [6].

It has been reported that radio frequency ablation (RFA) is an effective procedure for hepatocellular carcinoma (HCC) as well as for metastatic liver tumors [7,8]. However, it has also been shown that it is not uncommon for RFA to cause various complications [9,10]. During or just after the procedure, peritoneal bleeding, hepatic abscess, hemothorax, perforation of the gastrointestinal wall, and rapid hepatic decompensation can occur. In addition to these acute complications, intrahepatic recurrence occurs at a relatively high rate following RFA [11,12] and can appear as either a local recurrence or a multiple scattered recurrence. Seki et al. described a case of rapid progression of numerous tumors around the treated area after RFA for a small HCC [13]. Takada et al. and Nicoli et al. reported cases of bilobular multiple recurrence that occurred 6 months after RFA [14,15]. More recently, Ruzzenente et al. reported on patients with HCC who suffered from rapidly spreading recurrence after RFA, which was observed in 4.5% of patients [16]. We also reported the clinical study of scattered and rapid intrahepatic recurrences [17]. The common characteristics of these recurrences were rapid growth and scattered location, and they were found to occur around the ablated tumor or throughout the liver. We presumed that scattered recurrence could be attributable to an increase in intra-tumor pressure during ablation and a subsequent explosion of the ablated tumor. In a previous study using an *in vitro *porcine liver model [18], we demonstrated that the RFA procedure could produce an extreme increase in pressure. Because the scattered pattern of recurrence was associated with a poorer prognosis, we also developed a novel multi-step, incremental expansion (multi-step) using a modified expansion-type electrode technique, which was shown to result in significantly lower pressures. In addition to the LeVeen needle, the cool-tip needle (Radionics, Burlington, MA, USA), a non-expansion-type electrode, has been accepted clinically as a useful local treatment for HCC. In this study, we evaluated the maximal pressure during ablation and the total ablation time under the multi-step, single-step, or cool-tip method.

## Results

**Figure 1:**
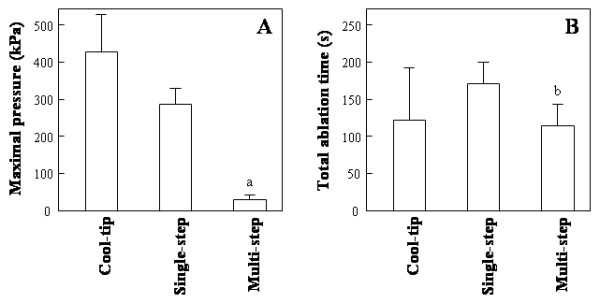
The maximal pressure (A) and total ablation time (B) on the porcine liver model, compared for the various tested methods. The multi-step method with a LeVeen needle resulted in a significantly lower pressure than the cool-tip procedure or the single-step method, and a total ablation time equal to that of the cool-tip procedure. All measurements were performed four times, and the results are expressed as mean ± SD. Statistical comparisons for the maximal pressure and the total ablation time were made using ANOVA and Scheffe's test. ^**a**^*p *< 0.05 *vs*. cool-tip; ^**b**^*p *< 0.05 *vs*. single-step.

As a model of an HCC tumor with a capsule, we prepared blocks of pig liver tissue packed into a rigid plastic case and the blocks were used in this study (see Methods). Figure 1A shows the peak pressure with the LeVeeen electrode (multi-step or single-step) and cool-tip electrode procedures (40 W ablation). With the LeVeen electrode procedure, the peak pressure produced during ablation was significantly lower than that with the cool-tip procedure. The peak pressure of 40 W was the highest among the various procedures; cool-tip 40 W, 416.3 ± 108.4 kPa > single-step method, 279.1 ± 29.6 kPa > multi-step method, 27.4 ± 13.8 kPa. The data are presented as mean ± standard deviation (SD).

In contrast, there was no significant difference in the ablation time between the multi-step (118.3 ± 16.4 s) and cool-tip ablation methods (123.7 ± 67.0 s), although the single-step method had longer ablation time (172.0 ± 26.9 s) than the other ablation procedures (Figure 1B). Although the multi-step method required ten times 'roll-off', the ablation was completed within less than 10 s before the fifth step. As a result, the cumulative ablation time was significantly shorter than that of the single-step method, and there was no significant difference in the total ablation time between the cool-tip (40 W) and multi-step methods.

In a preliminary clinical trial, we compared ablation time among the multi-step (n = 14), single-step (n = 13), and cool-tip (n = 13) methods. The multi-step method showed a significantly shorter ablation time than the single-step or cool-tip method (Table 1), and there was no significant difference in the area ablated by RF. Rapid and scattered recurrence after RFA occurred in some patients treated by the cool-tip or single-step method, but we found no cases of scattered recurrence associated with the multi-step procedure (Table 1). There were no other adverse events during treatment in the patients.

**Table 1:** Comparison of clinical backgrounds, ablation time, and RFA-treated "area" size among the single-step, multi-step, and cool-tip methods in clinical HCC cases.

	**LeVeen needle**		**Cool-tip needle**
		
	**Single-step**	**Multi-step**	
	
**Number**	13	14	13
**Sex (male/female)**	9/4	10/4	8/5
**Age**	55.8 ± 2.7	56.7 ± 2.5	54.1 ± 3.3
**Child A/Child B**	10/3	11/3	9/4
**Tumor location (r/l lobe)**	11/2	10/4	10/3
**Tumor size (mm)**	22.0 ± 2.3	21.5 ± 1.9	20.8 ± 3.1
**Ablation time (min)**	22.1 ± 1.9	10.9 ± 2.5 ^**b, c**^	15.4 ± 1.1 ^**b**^
**Ablated "area" (mm)**	32.1 ± 2.6	34.2 ± 2.7	35.8 ± 3.7
**Scattered recurrence^a^**	1	0	2

Tumor size and ablated area by RFA are expressed as the diameter (mm). χ^2^-test, ANOVA, and Scheffe's test showed no significant background difference among the single-step, multi-step, and cool-tip methods. ^**a **^Number of the patients in whom scattered intrahepatic recurrence occurred after radio frequency ablation (RFA). ^**b **^*p *< 0.001 *vs*. single-step; ^**c **^*p *< 0.01 *vs*. cool-tip.

## Discussion

In this study, we demonstrated that the multi-step method with a LeVeen electrode resulted in the lowest pressure as compared with the single-step or cool-tip method (Figure 1A). There was no significant difference in the ablation time between the multi-step and cool-tip ablation methods, although the single-step method had longer ablation times than the other ablation procedures (Figure 1B). The difference in pressure during the ablation is probably attributable to the differences in the region of ablation. With the cool-tip and single-step procedures, all ablated cells in the entire targeted region would expand simultaneously to generate high pressure, while the ablation of a limited region in the multi-step procedure would result in a lower level of intra-tumor pressure. In our previous report [18], based on histological findings, we concluded that the cell density of the internal region was higher than that of the outer region, and that some of the pressure caused by ablation during the second and subsequent steps could escape into the internal region.

Although an *in vitro *porcine liver model was accepted in this study, it may be necessary to confirm the phenomenon in an *in vivo *model because the impedance during ablation *in vitro *differs from that observed during the treatment of patients. Under the condition of porcine liver blocks covered entirely with hard plastic, the pressure near the ablated area during RFA increased to over 100 kPa, whereas the measured pressure in normal porcine liver *in vivo *was much lower in our previous study [19]. A substantial increase in pressure should be necessary for the tumor to explode during RFA. It is considered that, in normal liver *in vivo*, the pressure generated can easily escape to the surrounding parenchyma or blood vessels. On the other hand, the different conditions exist in most of HCC patients, a fibrotic capsule around the tumor and parenchymal fibrosis surrounding the tumor accompanied by cirrhosis. Therefore, during clinical RFA treatment for HCC in cirrhotic liver, the escape of pressure is more or less blocked and the pressure in the ablated area may possibly reach the level sufficient to cause an explosion. Although our *in vitro *pig liver block model is artificial and without blood flow, we assume the situation in our model to be that in a tumor with poor arterial flow and a thick capsule.

Because the reported scattered recurrence after RFA would be attributable to an increase in intra-tumor pressure during ablation and a subsequent explosion in the ablated tumor [13-16], for clinical application, our aim should be to reduce intra-tumor pressure during RFA. We showed that our multi-step method using a LeVeen needle resulted in much lower pressure than the cool-tip or the standard single-step method, both of which might entail a risk of extreme increase in intra-tumor pressure under some conditions. We should also aim to shorten the ablation time in order to reduce the patients' discomfort during treatment. We demonstrated that the multi-step procedure takes the same amount of time as the cool-tip method. We are now applying our multi-step method in clinical RFA treatment, and preliminary results indicated that the multi-step method consumed significantly shorter ablation times than the single-step or cool-tip method (Table 1). We will collect more clinical data in order to evaluate the appropriateness of this procedure for clinical use, and to confirm whether the intratumor pressure created by the sudden heating of RFA contributes to the spreading or local recurrence of HCC.

## Conclusion

Critical complication of rapid and scattered recurrence after RFA may possibly be avoided by the use of modified protocols. We consider that the multi-step method using a LeVeen needle may be one of the clinically applicable procedures for RFA.

## Methods

### Measurement of ablation time and pressure in vitro model

We measured the pressure in a block of porcine liver sealed in a rigid plastic case using a pressure sensor (model P303-01, M0101D; SSK. Co., Ltd., Tokyo, Japan) as previously reported [17]. Two blocks of liver tissue (5 × 5 × 4 cm) were cut and packed into a rigid 5 × 5 × 8 cm plastic case with the pressure sensor mounted at one end. We used two different systems, a LeVeen™ multipolar array needle (3.0 cm diameter type) in combination with an RF 2000 generator™ (Radio Therapeutics Corporation), and a Cool-tip™ RF System (3.0 cm exposure length type) (Radionics). The electrode needle was inserted from the opposite end of the apparatus to the pressure sensor, until the tip of the needle reached 3 cm from the sensor.

The LeVeen needle was used with either a standard protocol (single-step method) or a modified protocol (multi-step method). For the single-step method, the tines were fully expanded after the needle was inserted to the target position. RF energy was then applied to the tissue at an initial power setting of 40 W and was subsequently increased at increments of 10 W per minute to a maximum power of 75 W. The power setting was left at this point until power 'roll-off' occurred; tissue impedance (an increase in tissue resistance caused by decreased conductivity of electrical current due to protein denaturation and loss of intracellular fluids) rose to over 200, at which time the power passively decreased to less than 10 W. If no roll-off occurred, a total of 15 min elapsed. Using the same device, the multi-step method involved expanding one-tenth of the length of the electrode tines at first step, and the current was delivered until power roll-off occurred. At the second step, immediately following roll-off, two-tenths of the length of the tines was expanded and a current was supplied. With stepwise expansion of the tines, the ablation was repeated until the tines were fully expanded. RF energy was applied with an initial power setting of 30 W in first step. When power roll-off occurred within 30 s at a given step, the ablation at the next step was started at the same electrical power. If roll-off took more than 30 s, the next step was started with the power set 10 W up to 75 W. If the stepwise increase in power reached the maximum level before the final step, the ablation at the subsequent step was performed at maximum power. For the cool-tip electrode method, RF energy delivery was started at 40 W. The electric power was then increased by 10 W every minute. The maximum electrical power was 120 W, and the RF energy delivery was continued until the impedance increased beyond the limit of the generator.

### Measurement of ablation time in clinical HCC cases

We performed a preliminary comparison of ablation times for the single-step, multi-step, and cool-tip methods in 36 HCC cases with liver cirrhosis (Table 1). For the single-step method (n = 13), ablation was started at 50 W, and the electrical power was increased by 10 W per minute in the subsequent ablation until 90 W was reached. For the multi-step method (n = 14), ablation was started at 50 W. The electrical power was increased to 70 W at the fifth step and to 90 W at the final step. For the cool-tip method (n = 13), RF energy delivery was started at 40 W. The electric power was then increased by 10 W every minute. The maximum electrical power was 120 W, and the RF energy delivery was continued until the impedance increased beyond the limit of the generator. Tumor location, tumor size, and the area ablated by RFA were determined by computed tomography (CT) examination.

### Statistical analysis

Baseline characteristics of the patients prior to RFA treatment are shown as mean ± SD, and the statistical comparisons were performed using the χ^2^-test for categorical data and the non-paired *t*-test for numeric data. Regarding the *in vitro *model, all measurements were performed four times and the results are shown as mean ± SD. Statistical comparisons for the cumulative ablation time and the pressure at the programmed endpoint *in vitro *were made using ANOVA and the Scheffe's test, via the Statview software (SAS Institute, Cary, NC, USA).

## Competing interests

The author(s) declare that they have no competing interests.

## Authors' contributions

MN, ME, and HS participated in the experimental design, and performed most of the analyses and writing of the manuscript. MK, SM, TY, YM, and KK measured the ablation time and pressure. All authors read and approved the final manuscript.
